# Dynamical pathway analysis

**DOI:** 10.1186/1752-0509-2-9

**Published:** 2008-01-27

**Authors:** Hao Xiong, Yoonsuck Choe

**Affiliations:** 1 Department of Computer Science, Texas A&M University, College Station, TX 77843, USA

## Abstract

**Background:**

Although a great deal is known about one gene or protein and its functions under different environmental conditions, little information is available about the complex behaviour of biological networks subject to different environmental perturbations. Observing differential expressions of one or more genes between normal and abnormal cells has been a mainstream method of discovering pertinent genes in diseases and therefore valuable drug targets. However, to date, no such method exists for elucidating and quantifying the differential dynamical behaviour of genetic regulatory networks, which can have greater impact on phenotypes than individual genes.

**Results:**

We propose to redress the deficiency by formulating the functional study of biological networks as a control problem of dynamical systems. We developed mathematical methods to study the stability, the controllability, and the steady-state behaviour, as well as the transient responses of biological networks under different environmental perturbations. We applied our framework to three real-world datasets: the SOS DNA repair network in *E. coli *under different dosages of radiation, the GSH redox cycle in mice lung exposed to either poisonous air or normal air, and the MAPK pathway in mammalian cell lines exposed to three types of HIV type I Vpr, a wild type and two mutant types; and we found that the three genetic networks exhibited fundamentally different dynamical properties in normal and abnormal cells.

**Conclusion:**

Difference in stability, relative stability, degrees of controllability, and transient responses between normal and abnormal cells means considerable difference in dynamical behaviours and different functioning of cells. Therefore differential dynamical properties can be a valuable tool in biomedical research.

## Background

Cell functions are complex temporal processes and should be studied as complex dynamical processes rather than only in their individual steady states. It is increasingly recognized that it is the dynamics and the internal structures of the biological systems that give rise to the functioning of cells [1]. Currently, uncovering co-expressed genes and discovering differentially expressed genes are the primary methods for discovering the role of genes in disease pathogenesis [2], but these methods offer only static views and steady-state explanations and thus fail to account for the transient behaviours that influence phenotypes. Genetic regulatory networks seek to model complex interactions and dynamics of gene regulations. Genetic networks should behave differently in sick cells vs. healthy cells because genes that cause diseases behave fundamentally differently, and that difference should be reflected in their dynamical properties. Dynamical properties of genetic networks such as their response time have been studied mostly in the context of network motifs [3,4], but now we propose that they be investigated for their difference in normal vs. abnormal cells.

In this report we studied four dynamical properties: stability, relative stability, controllability, and transient behaviours (overshoot, settling time, and rise time). Stability governs how a system responds to internal noise and external perturbation and determines whether the system returns to steady states and whether the effect of noise and perturbation diminishes over time. Biologically, an unstable cellular system is very brittle and the slightest disturbance can drive the system beyond tolerance and possibly result in cell death. Prill et al. [5] used stability as a criterion to discern network motifs and their organizing principles, and synthetic biologists are beginning to pay close attention to the stability of their artificial networks [6]. Furthermore, the stability of the system under pure gain feedback control can be analyzed by the root-locus method and the result can be interpreted as a measure of relative stability. In control theory, the root-locus method is a design tool but it is also used as an analytic tool, to see how large a gain can drive the system unstable with feedback loops: the larger margins of stabilizing gains, the better. Related to feedback control, controllability is another pivotal concept in control theory. It and its dual property, observability, were originally conceived as solutions to existence and uniqueness problems of optimal control [7], and the controllability of a dynamical system roughly refers to the ability to move the states of the system around the state space with reasonable efforts. Although controllability is a binary question, there is a measure of the degree of controllability, the idea being that the more controllable a system is the less effort is needed to move the system. Less theoretical than stability and controllability are transient behaviours like settling time and overshoots, which have also received attention from systems biologists [3,4,8]. These four dynamical properties are determined by the parameters of the dynamical system and the unknown parameters of biological systems need to be estimated.

Parameter estimation must be done under a particular modelling framework. Several modelling frameworks have been proposed: Boolean networks [9-12], differential equations [13], S-system [14,15], and dynamical Bayesian networks [16,17]. A special case of dynamical Bayesian networks is the state-space model, which has been used to model genetic regulatory networks [18-22]. A state-space model has states, inputs, and outputs, where hidden states contain complete information of the system driven by the inputs, and the outputs are the measurements made by scientists. In the state-space models of genetic networks, states are the regulatory elements, and the inputs and the outputs can be environmental stimuli or expression levels. Because genetic networks have many unknown quantities, state-space models can serve as a good modelling framework.

In this paper, the parameters in state-space models were estimated from the time course of gene expressions using Kalman filter and the constrained expectation-maximization (EM) algorithm (a modified EM algorithm that incorporates prior knowledge about the structure of genetic networks). The regular EM algorithm is commonly used to estimate parameters in the presence of hidden quantities, and they comprise two steps, E-step (expectation) and M-step (maximization), where the E-step estimates the hidden states, and the M-step the parameters [23]. We applied EM algorithm to three sets of time course data and estimated three genetic networks for analysis.

**Figure 1:**
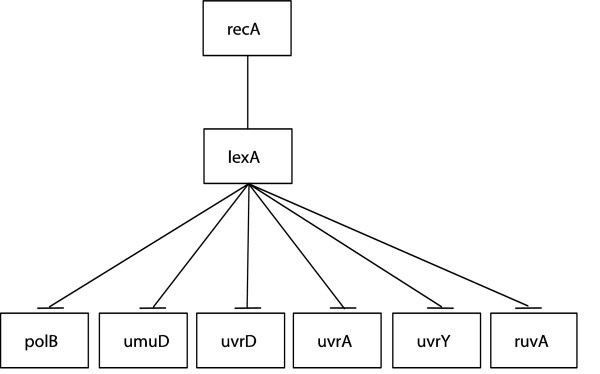
The diagram of SOS DNA repair network.

**Figure 2:**
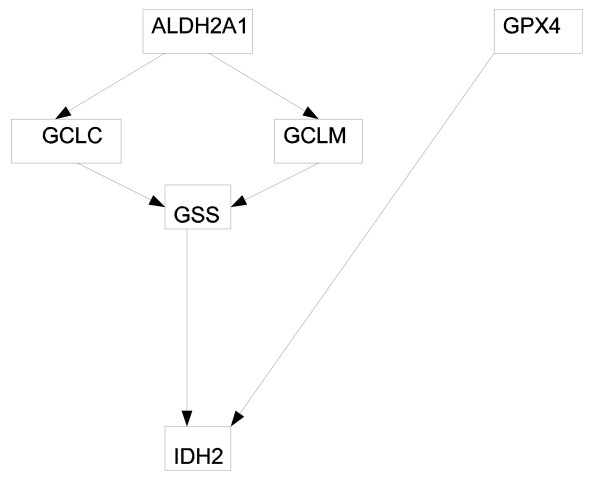
The diagram of GSH redox cycle.

**Figure 3:**
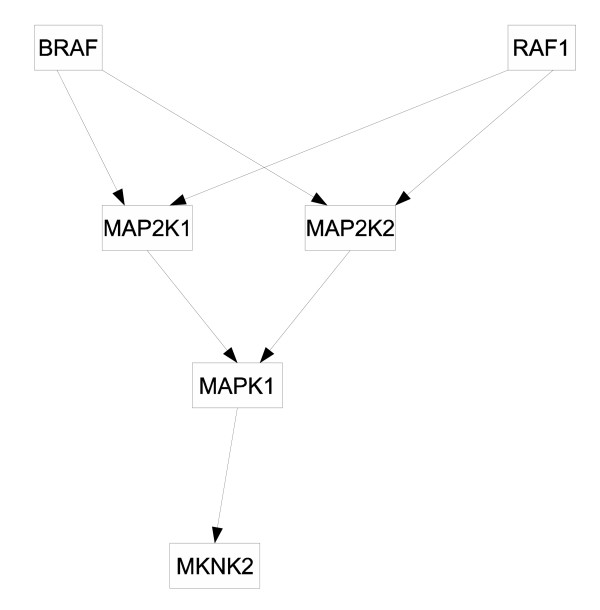
The diagram of MAPK network.

The first network we used is the SOS DNA repair system. The SOS network is a highly conserved system [8,24] and consists of about 30 genes, the master regulator being gene lexA. The lexA gene inhibits the rest of the SOS network's genes under normal conditions, but when DNA damage is sensed, protein LexA is cleaved and the genes normally suppressed are activated. A diagram of the SOS network with 8 essential genes is shown in Fig. 1. Shown in Fig. 2 is the second system we modelled, the glutathione (GSH) redox cycle with one gene from the urea cycle that interacts with the redox cycle [25]. The data are from Sciuto et al. [26] who investigated the differential gene expressions in mice lung cells exposed to either carbonyl chloride (phosgene) or normal air. They found elements of the GSH redox cycle differentially expressed, which is not surprising given that the redox cycle is heavily involved in protecting organisms from reactive oxygen species, that it is heavily present in the lung, and that phosgene causes massive lung damages. The third system we investigated is the mitogen-activated protein kinase (MAPK) network in cell lines disturbed by either the wild type HIV type I Vpr or the mutant type R73A or the mutant type R80A. HIV-1 Vpr is an important protein in promoting the pathogenesis of AIDS by facilitating apoptosis and cell cycle stall at G2. Yoshizuka et al. [27] studied the effects of Vpr on MAPK-network-related genes in stalling cell cycle, so they obtained cell lines that can express wild type or mutant Vpr under a tetracycline-inducible promoter. They found that many genes related to the MAPK network differentially expressed when subjected to different types of Vpr. The MAPK network used for this report is shown in Fig. 3. All those data sets compare the organism's reactions to different environmental perturbations, and from estimated genetic networks we hope to discover the differential dynamical properties of genetic networks under stress.

We applied our framework to three real-world time series datasets above and found differential stability, transient responses, and controllability of genetic networks in normal vs. abnormal cells.

## Results

### Models of genetic networks and their application to real data sets

We modeled genetic networks as dynamical systems, more specifically as linear state-space systems. A linear state-space model of dynamical systems can be written as

$$\begin{array}{c} x(t+1)=Ax(t)+Bu(t)+w\\ y(t)=Cx(t)+Du(t)+v \end{array}$$

where *x*(*t*) is the state vector, *y*(*t*) the output vector, and *u*(*t*) the input vector, all at time *t*; *w *and *v *are independent noise terms assumed to be white Gaussian with zero means and covariance *Q *and *R*, respectively. Matrix *A *is called the state transition matrix, *B *the input matrix, *C *the output matrix, and *D *the feed-forward matrix. Matrices *A*, *B*, *C*, *D *and covariance matrices *Q *and *R *together make up the parameters of the dynamical system.

The states represent the biological forces that regulate gene regulation; they describe the behaviours of gene transcription but are hidden. The outputs denote the gene expression levels and are measured, and it is assumed that the expression level of a gene is determined by the state of the regulated gene. The inputs can be any external stimuli that influence gene regulation: substances like drugs, proteins, RNAs, or expression levels of other genes.

### Estimated system

**Figure 4:**
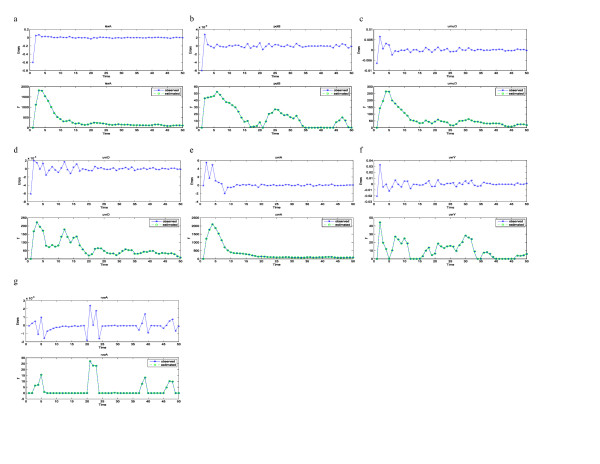
**Estimated expression levels and error in estimation**. For each gene of the SOS system, we have superimposed the estimated expression levels on measured expression levels and plotted the error in estimation in a separate panel. We have done this for the low radiation level data set in Figure 4. The estimations generally show good behaviors. Figs. 4a, 4b, 4c, 4d, 4e, 4f, 4g are for the low radiation level data set, and plotted genes lexA, polB, umuD, uvrD, uvrA, uvrY, and ruvA, respectively. Each gene has two plots; the bottom panel shows estimated expression level superimposed on measured expression level, while the top panel is the estimation error.

**Figure 5:**
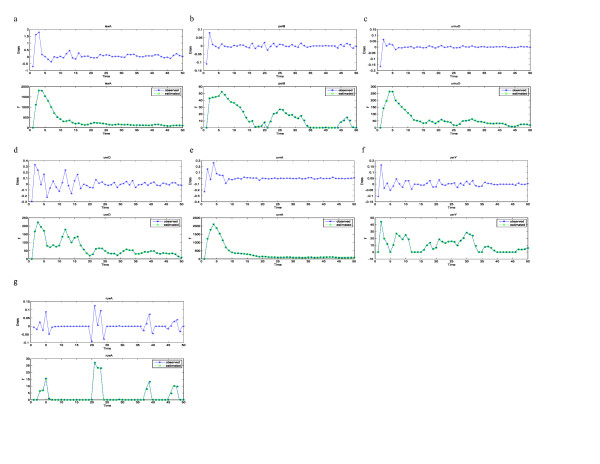
**Estimated expression levels and error in estimation**. We have superimposed the estimated expression levels on measured expression levels and plotted the error in estimation in a separate panel for the high radiation level data set in Figure 5. Figs. 5a, 5b, 5c, 5d, 5e, 5f, 5g are for the high radiation level data set plotted genes lexA, polB, umuD, uvrD, uvrA, uvrY, and ruvA, respectively. Each gene has two plots; the bottom panel shows estimated expression level superimposed on measured expression level, while the top panel is the estimation error.

For the SOS system, *x*_2 _is the discretized first derivative of *x*_1_, whereas *x*_1 _is the expression level of gene lexA, *x*_3 _gene polB, *x*_4 _gene umuD, *x*_5 _gene uvrD, *x*_6 _gene uvrA, *x*_7 _gene uvrY, and *x*_8 _gene ruvA. The outputs are the measured expression levels of the seven genes listed above, and the input is gene recA. In Fig. 4 and Fig. 5, we included the estimated outputs and the measured outputs superimposed into one plot, as well as estimation errors in a separate panel for each gene. From the plots we can see that the estimated trajectory largely follows measured values. The estimated system parameters are listed below for the low level of radiation:

$$\begin{array}{ll} x_{1}(t+1)=x_{1}(t)+x_{2}(t) & y_{1}(t)=x_{1}(t)\\ x_{2}(t+1)=-0.17x_{1}(t)+0.59x_{2}(t)+0.084u(t) & y_{2}(t)=x_{3}(t)\\ x_{3}(t+1)=0.009x{\left(t\right)}_{1}+0.81x_{3}(t) & y_{3}(t)=x_{4}(t)\\ x_{4}(t+1)=0.037x_{1}(t)+0.74x_{4}(t) & y_{4}(t)=x_{5}(t)\\ x_{5}(t+1)=-0.007x_{1}(t)+0.964x_{5}(t) & y_{5}(t)=x_{6}(t)\\ x_{6}(t+1)=-0.037x_{1}(t)+0.965x_{6}(t) & y_{6}(t)=x_{7}(t)\\ x_{7}(t+1)=0.008x_{1}(t)+0.697x_{7}(t) & y_{7}(t)=x_{8}(t)\\ x_{8}(t+1)=0.009x_{1}(t)+0.621x_{8}(t)\text{.} & \end{array}$$

For the high level of radiation, the estimated system is

$$\begin{array}{ll} x_{1}(t+1)=x_{1}(t)+x_{2}(t) & y_{1}(t)=x_{1}(t)\\ x_{2}(t+1)=-0.242x_{1}(t)+0.329x_{2}(t)-0.014u(t) & y_{2}(t)=x_{3}(t)\\ x_{3}(t+1)=0.008x_{1}(t)+0.832x_{3}(t) & y_{3}(t)=x_{4}(t)\\ x_{4}(t+1)=0.051x_{1}(t)+0.653x_{4}(t) & y_{4}(t)=x_{5}(t)\\ x_{5}(t+1)=0.01x_{1}(t)+0.889x_{5}(t) & y_{5}(t)=x_{6}(t)\\ x_{6}(t+1)=-0.366x_{1}(t)+1.22x_{6}(t) & y_{6}(t)=x_{7}(t)\\ x_{7}(t+1)=0.002x_{1}(t)+0.906x_{7}(t) & y_{7}(t)=x_{8}(t)\\ x_{7}(t+1)=0.002x_{1}(t)+0.906x_{7}(t). & \end{array}$$

For the GSH redox cycle there are two inputs, gene ALD2A1 as *u*_1 _and GPX4 as *u*_2_. All the states were modelled with second order dynamics so the last four states *x*_5_, *x*_6_, *x*_7 _and *x*_8 _are the discretized first derivatives of *x*_1_, *x*_2_, *x*_3 _and *x*_4_, respectively. Here, gene GCLC is *x*_1_, gene GCLM *x*_2_, gene GSS *x*_3_, and gene IDH2 *x*_4_. The estimated system for exposure to normal air is

$$\begin{array}{ll} x_{1}(t+1)=x_{1}(t)+x_{5}(t) & \\ x_{2}(t+1)=x_{2}(t)+x_{6}(t) & \\ x_{3}(t+1)=x_{3}(t)+x_{7}(t) & y_{1}(t)=x_{1}(t)\\ x_{4}(t+1)=x_{4}(t)+x_{8}(t) & y_{2}(t)=x_{2}(t)\\ x_{5}(t+1)=-0.37x_{1}(t)-0.39x_{5}(t)+0.814u_{1}(t) & y_{3}(t)=x_{3}(t)\\ x_{6}(t+1)=-0.429x_{2}(t)-0.006x_{6}(t)+0.632u_{2}(t) & y_{4}(t)=x_{4}(t)\\ x_{7}(t+1)=0.095x_{1}(t)-0.015x_{2}(t)-0.217x_{3}(t)-0.128x_{7}(t) & \\ x_{8}(t+1)=0.753x_{3}(t)-0.409x_{4}(t)-0.867x_{8}(t)+0.017u_{2}(t) & \end{array}$$

For exposure to phosgene, the estimated model is

$$\begin{array}{ll} x_{1}(t+1)=x_{1}(t)+x_{5}(t) & \\ x_{2}(t+1)=x_{2}(t)+x_{6}(t) & \\ x_{3}(t+1)=x_{3}(t)+x_{7}(t) & y_{1}(t)=x_{1}(t)\\ x_{4}(t+1)=x_{4}(t)+x_{8}(t) & y_{2}(t)=x_{2}(t)\\ x_{5}(t+1)=-0.141x_{1}(t)+1.95x_{5}(t)+0.77u_{1}(t) & y_{3}(t)=x_{3}(t)\\ x_{6}(t+1)=-0.076x_{2}(t)-1.09x_{6}(t)-0.13u_{2}(t) & y_{4}(t)=x_{4}(t)\\ x_{7}(t+1)=0.05x_{1}(t)-0.161x_{2}(t)-0.09x_{3}(t)-0.705x_{7}(t) & \\ x_{8}(t+1)=0.336x_{3}(t)-0.179x_{4}(t)-1.126x_{8}(t)+0.103u_{2}(t). & \end{array}$$

As for the MAPK system, the inputs are gene BRAF as *u*_1_and gene RAF1 as *u*_2_. The states *x*_1_, *x*_2_, *x*_3 _and *x*_4 _are genes MAP2K1, MAP2K2, MAPK1, and MKNK2, respectively; the other four states are the discretized first derivatives as in the system for the GSH redox cycle. The estimated system for the wild type Vpr is

$$\begin{array}{ll} x_{1}(t+1)=x_{1}(t)+x_{5}(t) & \\ x_{2}(t+1)=x_{2}(t)+x_{6}(t) & \\ x_{3}(t+1)=x_{3}(t)+x_{7}(t) & y_{1}(t)=x_{1}(t)\\ x_{4}(t+1)=x_{4}(t)+x_{8}(t) & y_{2}(t)=x_{2}(t)\\ x_{5}(t+1)=-1.48x_{1}(t)-1.32x_{5}(t)+0.14u_{1}(t)+0.2u_{2}(t) & y_{3}(t)=x_{3}(t)\\ x_{6}(t+1)=-0.098x_{2}(t)-0.52x_{6}(t)-0.079u_{1}-0.314u_{2}(t) & y_{4}(t)=x_{4}(t)\\ x_{7}(t+1)=0.498x_{1}(t)+0.052x_{2}(t)-0.215x_{3}-0.618x_{7}(t) & \\ x_{8}(t+1)=0.123x_{3}(t)-0.169x_{4}(t)-0.602x_{8}(t), & \end{array}$$

and for the R73A mutant

$$\begin{array}{ll} x_{1}(t+1)=x_{1}(t)+x_{5}(t) & \\ x_{2}(t+1)=x_{2}(t)+x_{6}(t) & \\ x_{3}(t+1)=x_{3}(t)+x_{7}(t) & y_{1}(t)=x_{1}(t)\\ x_{4}(t+1)=x_{4}(t)+x_{8}(t) & y_{2}(t)=x_{2}(t)\\ x_{5}(t+1)=-1.098x_{1}(t)-1.087x_{5}(t)+0.068u_{1}(t)-0.23u_{2}(t) & y_{3}(t)=x_{3}(t)\\ x_{6}(t+1)=-0.76x_{2}(t)-x_{6}(t)-0.06u_{1}(t)-0.03u_{2}(t) & y_{4}(t)=x_{4}(t)\\ x_{7}(t+1)=0.073x_{1}(t)+0.5x_{2}(t)-0.355x_{3}(t)-0.647x_{7}(t) & \\ x_{8}(t+1)=0.79x_{3}(t)-0.876x_{4}(t)-1.179x_{8}(t)\text{,} & \end{array}$$

and for R80A mutant

$$\begin{array}{ll} x_{1}(t+1)=x_{1}(t)+x_{5}(t) & \\ x_{2}(t+1)=x_{2}(t)+x_{6}(t) & \\ x_{3}(t+1)=x_{3}(t)+x_{7}(t) & y_{1}(t)=x_{1}(t)\\ x_{4}(t+1)=x_{4}(t)+x_{8}(t) & y_{2}(t)=x_{2}(t)\\ x_{5}(t+1)=-0.582x_{1}(t)-0.821x_{5}(t)+0.082u_{1}(t)-0.085u_{2}(t) & y_{3}(t)=x_{3}(t)\\ x_{6}(t+1)=-0.28x_{2}(t)-0.836x_{6}(t)-0.149u_{1}(t)-0.009u_{2}(t) & y_{4}(t)=x_{4}(t)\\ x_{7}(t+1)=-0.019x_{1}(t)+0.273x_{2}(t)-0.056x_{3}(t)-0.249x_{7}(t) & \\ x_{8}(t+1)=0.112x_{3}(t)-0.467x_{4}(t)-1.248x_{8}(t)\text{.} & \end{array}$$

Although the number of parameters is small compared with the number of states, which agrees with the knowledge that genetic networks are sparse [28], it is still hard to see at a glance whether they differ in any fundamental way. For that, we must apply systematic analysis to the estimated systems.

### Differential stability of systems under different perturbations

Stability is a very important property of a biological system, for an unstable system puts great stress on neighbouring systems and may even lead to cell death. A system is stable if it will converge to steady states after disturbance; it is unstable otherwise. The stability of a discrete linear system can be determined by the eigenvalues of its state transition matrix *A*: if all the eigenvalues are within the unit circle in the complex plane, then the discrete system is stable. The eigenvalues of the three analyzed networks are listed in Table 1, 2, and 3, and their implications discussed below.

**Table 1:** Differential stability of the SOS network

Low Dosage	High Dosage
0.8117	0.8321
0.7367	0.6530
0.9637	0.8893
0.9652	**1.2216 (unstable)**
0.6969	0.9062
0.6219	0.6291
0.7952 + 0.3630i	0.6647 + 0.3597i
0.7952 - 0.3630i	0.6647 - 0.3597i

**Table 2:** Differential stability of GSH redox cycle by

Normal Air	Phosgene
-0.6141	**2.0830 (unstable)**
0.1177	**-1.0383 (unstable)**
0.4803 + 0.3199i	-0.7561
0.4803 - 0.3199i	**1.0512 (unstable)**
0.7467	0.9120
0.7542	0.8696
0.4972 + 0.4196i	1.0470 + 0.2711i
0.4972 - 0.4196i	1.0470 - 0.2711i

**Table 3:** Differential stability of the MAPK network

Wild type	R73A	R80A
0.8527	0.7448	**-1.0169 (unstable)**
0.8862	-0.0437 + 0.0925i	-0.4078
-0.1615 + 0.3646i	-0.0437 - 0.0925i	-0.2023
-0.1615 - 0.3646i	-0.6472	0.5867
-0.4884	-0.3913	0.9534
-0.4601	0.4680	0.7685
0.9324	0.4877	-0.6676
-0.4477	-0.4916	0.8315

We analyzed the SOS DNA network under low and high dosage of radiation and discovered that the network was stable for low dosage and unstable for high dosage. We found that the eigenvalues of SOS network under low dosage of radiation to have the eigenvalues' norm all less than one, and therefore the network was stable. On the other hand, the SOS network was unstable under high dosage of radiation, as the norm of one of its eigenvalues was greater than one.

We also analyzed the redox cycle in mice lung cells that were exposed to either carbonyl chloride (phosgene), an industrial toxin, or normal air; and we found that GSH redox system in normal lung cells was stable – all eigenvalues were within the unit circle, and that the network exposed to toxin was unstable – some eigenvalues were outside the unit circle. Whether the unstable detoxification system contributed to the death of mice exposed to phosgene is not yet known, but Sciuto et al. [26] speculated that the poison might have overwhelmed the detoxification system.

We also analyzed the activity data from the MAPK network in mammalian cells that expressed either wild type Vpr, mutant R73A Vpr, or mutant R80A Vpr; and we found that both the wild type and R73A produced stable behaviours, and R80A caused the network to become unstable. A stable MAPK network helps the virus most, for Yoshizuka et al. [27] found the HIV virus uses MAPK network to cause cell cycle G2 arrest, and over-expression of MAP2K2 reversed the arrest.

### Differential relative stability analyzed via root locus

The relative stability of genetic networks is also important; it is a measure of robustness. We studied relative stability by examining the stability margins of pure gain feedback loops through root-locus plots. Given a dynamical system, one forms a feedback loop from the output to the input through only a pure gain controller. Depending on whether the control signal is negated as it is fed into the input, the feedback can be positive (not negated) or negative (negated). The original system is called the open-loop system, and its zeros and poles are the open-loop zeros and poles; the zeros and poles of the overall system are called the closed-loop zeros and poles. A dynamical system's zeroes are the roots of the numerator of the transfer function (for an explanation of the transfer function, see Methods), and the poles are the roots of the denominator. The stability of closed-loop systems depend on the closed-loop poles. The root-locus method generates a plot that traces the closed-loop poles as the gain of the controller is varied, and the portion of gains that make the closed-loop stable is called the stability margin. In the root locus plot, the open-loop zero is represented by a circle (○), the open-loop pole by a cross (×), and if there is a zero-pole cancellation we will see a circle and a cross on top of each other (⊗). The root-locus method can only study systems with single input and single output (SISO), but the dynamical properties of SISO systems is a reflection of the overall system's dynamical properties, so that the performance of the SISO system will manifest itself in the overall system's performance.

**Figure 6:**
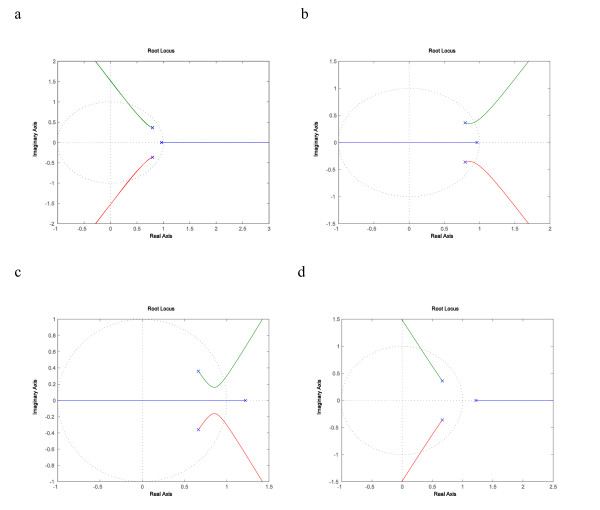
**Root locus plots of recA to uvrA SISO system**. These plots trace the poles of the closed-loop system as the gain *K *is varied from zero to infinity. The trajectories start at the open-loop poles which are represented by the cross, and could end at the open-loop zeros which are represented by an open circle, or they could go on infinitely in some direction. The different colours represent distinct trajectories of different closed-loop poles. Fig. 6a This is the root locus plot of recA to uvrA system under low level of radiation with negative feedback, where the locus on the real axis goes out of the unit circle quickly and therefore shows small stability margins. (The dotted circle is the unit circle.). Fig. 6b This is the root locus plot of recA to uvrA system under low level of radiation with positive feedback, with some stability margins. Fig. 6c This is the root locus plot of recA to uvrA system under high level of radiation with negative feedback, where a good portion of all three loci stays within the unit circle and therefore exhibits large stability margins. Fig. 6d This is the root locus plot of recA to uvrA system under high level of radiation with positive feedback, which has no stability margin.

In the SOS DNA repair network, the recA to uvrA SISO system showed differential root-locus plots, depending on radiation levels. Their respective root-locus plots, for both negative and positive feedbacks, are shown in Fig. 6a, 6b, 6c, and 6d. Under low level of radiation, we found that the SISO system was comfortable with positive feedback, which had larger margin of stabilizing gains, whereas negative feedback allowed far narrower choices. Under high level of radiation, the opposite was true: positive feedback had no stabilizing gain whereas negative feedback had a large margin. The need for positive feedback loop in low radiation level is an interesting discovery from our root-locus analysis, because it runs counter to the common perception that negative feedback loop promotes stability and positive feedback loop leads to instability. Perhaps under low radiation level, the SOS network is not sufficiently stimulated and positive feedback fully activates the network which then leads to overall stability.

**Figure 7:**
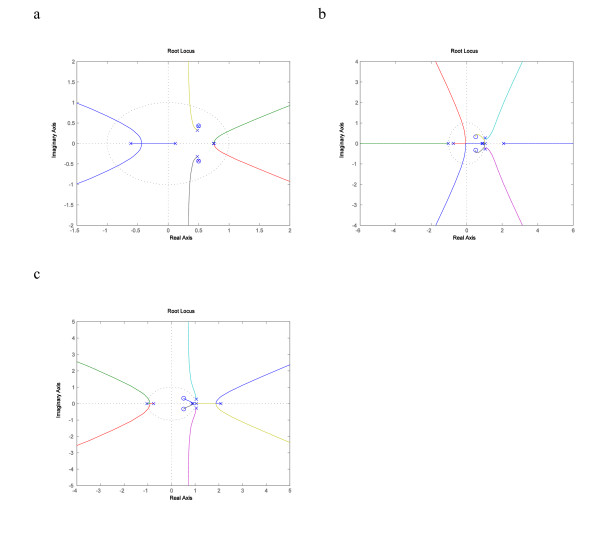
**Root locus plots of recA to uvrA SISO system**. Fig. 7a Root locus plot of ALDH2A1 to IDH2 system exposed to normal air with negative feedback is shown, with large stability margins. Fig. 7b Root locus plot of ALDH2A1 to IDH2 system exposed to poisonous air with negative feedback, where the locus on the positive real axis is entirely outside of the unit circle and therefore it has no stability margin. Fig. 7c Root locus plot of ALDH2A1 to IDH2 system exposed to poisonous air with positive feedback, showing no stability margin because of the locus at the right.

In the GSH redox network, we discovered that the ALDH2A1 to IDH2 SISO system showed a simpler but more striking difference under different environmental conditions. When exposed to normal air, the SISO system was stable and the root-locus plot in Fig. 7a shows that sizeable gain values do not destabilize the closed-loop system, which represents a nice scenario, because the subsystem can sustain a lot of stress. But, as we can see in Fig. 7b and Fig. 7c, the same SISO system, when exposed to toxin, not only had an unstable open-loop system, but the closed-loop system also remained unstable no matter what value of the gain was, positive or negative. This means that not only the ALDH2A1 to IDH2 SISO system was very unstable, but that a higher order controller must be used to produce a stable closed-loop system, a sign of very serious damage.

**Figure 8:**
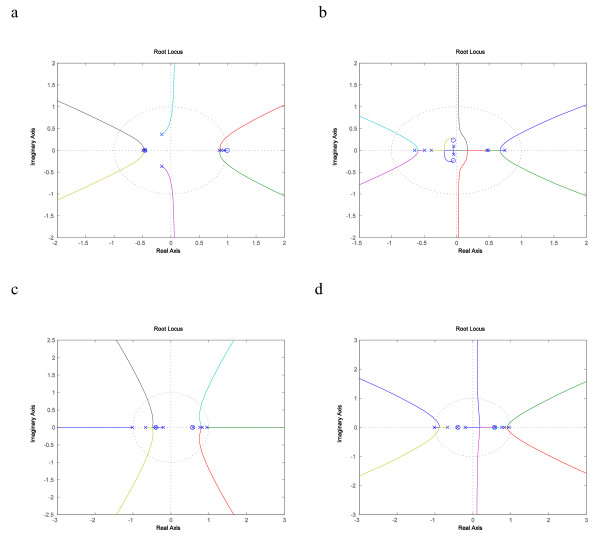
**Root locus plots of RAF1 to MKNK2 SISO system**. Fig. 8a Root locus plot of RAF1 to MKNK2 system perturbed by wild type Vpr with negative feedback where a large portion of the locus can be seen within the unit circle. Fig. 8b Root locus plot of RAF1 to MKNK2 system perturbed by R73A mutant Vpr with negative feedback showing very good stability margins. Fig. 8c Root locus plot of RAF1 to MKNK2 system perturbed by R80A mutant Vpr with negative feedback, where there is basically no stability margin due to the two loci on the real axis. Fig. 8d Root locus plot of RAF1 to MKNK2 system perturbed by R80A mutant Vpr with positive feedback and small stability margins.

We also found that MAPK network in mammalian cell lines subject to different versions of Vpr of HIV type I virus had similarly different root locus plots, which are shown in Fig. 8a, 8b, 8c, and 8d. The RAF1 to MKNK2 SISO system was stable under both the wild type and the R73A mutant Vpr perturbation, and both showed comfortable margin of gain values for which the closed-loop system was stable. The SISO system under R80A mutant protein exhibited a stable closed-loop system with only a small margin of gain with positive feedback and none with negative feedback. If that small margin does not include a gain that can produce a closed system with satisfactory performance, then a higher order controller is called for.

### Differential degree of controllability

Since one goal of systems biology is to aid the development of therapeutic treatments, which in the context of genetic networks is to bring the network from undesirable states to healthy states by manipulating inputs, the relative ease of moving around in the state space is an important issue. The ability to move a system from one point in the state space to another in finite time with only finite inputs is called controllability, which is a pivotal concept in linear time systems theory [7]. Controllability can be tested by the rank of controllability matrix; if the controllability matrix is of full rank, then the system is controllable, otherwise uncontrollable. Beyond the binary test (controllable or not) there are also degrees of controllability. The condition number of the controllability matrix can be considered as a measure of the degree of controllability, the bigger the number the less the controllability. A system with less controllability may require much greater inputs to achieve the desired final state, which could be a problem as the inputs for biological systems are drugs, radiation therapy, things in limited supply and subject to cost factors. As we will see, different systems could have radically different degrees of controllability.

Although we found all the three genetic regulatory networks controllable under all circumstances, their condition numbers differed, for one significantly. We discovered that the SOS DNA repair system under high dose of radiation had a condition number of 2.8·10^9 ^for its controllability matrix, and that under low dosage the condition number was 2.6·10^9^. The similarly large condition numbers suggest the SOS system under study is difficult to control; whether this is due to radiation is not known. On the other hand, in mice lung exposed to normal air we saw that the redox system had a condition number of 567 for its controllability matrix, and that those exposed to toxin had 70267. The different condition numbers peg the redox system as much more difficult to control after exposure to poison, perhaps due to damages or the fact that the network was being overwhelmed by the effects of the toxin. The third network, the MAPK network in mammalian cell lines, was found to have a condition number of 62.15 when exposed to the wild type Vpr, 88.5 for those exposed to the R80A mutant, and 285.4 for the R73A mutant. It is obvious that R73A mutant results in a stodgier MAPK network than other variants, but overall the MAPK system retains good controllability, making it a good target for treatment.

### Differential transient responses

To study cell functions as temporal processes means we must take stock of transient behaviors in addition to steady states. One way to characterize transient behaviors is through the transient response of the dynamical system to inputs like step input and impulse input, but because the step responses and impulses responses give same information for linear systems, we will concentrate on the step input responses. A step input is a constant input, a unit step, a constant unity. The rise time is a measure of the speed of the dynamics, and the settling time and the overshoot gauge how close to the steady state the transient behaviors are. Of course, systems that exhibit differential stability will have different transient responses, but because differential stability is addressed earlier, we will disregard any difference in transient responses due to stability difference.

The transient responses are by their nature studied as input-output pairs, also called a single-input-single-output (SISO) system. Although we will look at individual SISO system extracted from multiple-input-multiple-output (MIMO) systems, the transient responses are still the intrinsic properties of the original system, and differential transient responses suggest fundamentally different dynamical behaviors of the original system in response to external perturbations.

The SOS DNA repair network has only one SISO system, besides those due to differential stability, that exhibited differential rise time and settling time, the recA to uvrD system. The SISO system, when exposed to high radiation dosage, was almost twice as fast as the system exposed to low dosage of radiation, to reach their respective steady states. This suggests that the SOS system needs uvrD to respond faster to, and therefore has faster dynamics under, higher levels of radiation. With no overshoot in both cases and a smaller settling time for a higher dosage, the recA to uvrD system under high radiation level stayed closer to the steady states. The rise time and settling time are listed in Table 4.

**Table 4:** Different transient responses of the SOS network

	recA to uvrD
Low Radiation Dosage	RiseTime: 58.1993
	SettlingTime: 108.1459
High Radiation Dosage	RiseTime: 17.9888
	SettlingTime: 35.8853

The MAPK network in mammalian cells exhibited differential transient responses to three types of Vpr of HIV type I virus. The BRAF to MAP2K2 SISO system displayed slower dynamics and were more distant from the steady state under the wild type than both mutants, and among the mutants, R73A had faster dynamics and better ability to stay close to the steady state. On the other hand, the BRAF to MAPK1 system's transient behaviors in response to the wild type Vpr were dominated by a 440% overshoot, and with its long settling time the system's transient responses were far from the steady state. The system perturbed by the wild type protein also had faster dynamics due to its smaller rise time, and the R73A mutant produced a system that had relatively fast dynamics and transient response closer to the steady state. The R80A mutant resulted in a system with slow dynamics and transient responses distant from the steady state with its relative large rise time and settling time and no overshoot. The respective rise time, settling time, and overshoots are in Table 5.

**Table 5:** Different transient responses of the MAPK network

	BRAF to MAP2K2	BRAF to MAPK1
Wild type	RiseTime: 31.5	RiseTime: 0.26
	SettlingTime: 56.5	SettlingTime: 76.1
	Overshoot: 0	Overshoot: 440.4
R73A Mutant	RiseTime: 2.6	RiseTime: 8.2
	SettlingTime: 5.8	SettlingTime: 17.0
	Overshoot: 0	Overshoot: 0
R80A Mutant	RiseTime: 11.7	RiseTime: 48.5
	SettlingTime: 21.7	SettlingTime: 90.2
	Overshoot: 0	Overshoot: 0

Although overshoot is generally considered undesirable in engineering (whether fast dynamics or staying close to the steady states are good or bad depends on the circumstances and cannot be determined a priori;) a large overshoot can be a fast way of signalling, or it can be an unbearable disturbance to cells. But being aware of the difference in transient responses is the first step toward devising treatment strategies that shape biological systems' dynamics to our liking.

## Discussion

Discovering differentially expressed genes and clustering co-expressed genes into functional groups have given researchers hints about the role of genes in pathogenesis. However, with increasing recognition that cell functions are temporal processes and that the dynamics of gene expression levels and gene interactions play a vital role in determining the health of the organism [1,29], there is a need to distinguish peculiar dynamical behaviors that result in sickness from those that do not. Dynamical properties succinctly characterize dynamical behaviors, and differential dynamical properties of gene networks can be seen as a natural extension of differentially expressed genes.

In this report we analyzed the dynamical properties of genetic networks, such as their stability, their closed-loop stability embodied in the root-locus plot, their step responses, and their controllability. First, we estimated the state-space models of three genetic networks: the SOS DNA repair network, the GSH redox cycle system, and the MAPK network; then we performed analysis on the estimated models. From the preliminary results, we found that significant differences in dynamical properties exist in all three networks.

All three genetic networks exhibited differential stability. Stability is a fundamental dynamical property in any dynamical system. A dynamical system is unstable if it diverges or oscillates after being subjected to perturbations. An unstable system is sensitive to perturbation or noise, and it will have erratic behaviors, possibly causing irreparable cell damage, leading to impairment of cell functions and maybe even cell death. A stable genetic network on the other hand confers a degree of robustness against noise on the overall organism. Recently Hornstein and Shomron [30] proposed that miRNAs play a stabilizing role for a number of genetic networks and the stability was necessary for the proper functioning of organisms. It would be interesting to see whether restoring stability to an organism's genetic networks restores the organism's health.

Besides stability, we also studied relative stability. Root-locus plots track the stability of the closed-loop system under the influence of a pure gain controller for single-input-single-output (SISO) systems, and they can be seen as a measure of the relative stability of the SISO system. As biological systems are often under control of other, bigger systems, wide margins of stabilizing gains give more leeway to, and can sustain some stress from, the controlling systems, and therefore they are more relatively stable than those with narrow margins. The redox cycle system in mice lungs is the clearest example. Exposed to normal air, the ALDH2A1 to IDH2 system was itself stable and the closed-loop system was stable for all possible gains, which makes this SISO system robust in normal tissues. But when exposed to toxin, not only was it unstable in itself, but no gain value could make the closed-loop system stable, which makes the system brittle. Systems that change from high relative stability to low relative stability can be considered for association with diseases, because they impact the outer loop systems and could make the overall system unstable. However, relative stability is not the only thing root-locus plots can show. In the recA to uvrA SISO system of the SOS network, positive feedback enabled a lot of stabilizing gains for the SISO system exposed to low level of radiation, as opposed to the same system exposed to high level of radiation, which needed negative feedback for large margins of stabilizing gains. This may portend drastic changes in the outer loops, as changing from promotion to inhibition is not easy for biochemical reactions, and it could be a major sign that this system is associated with unhealthy conditions.

The last dynamical property we looked at was controllability. Therapeutic treatments can be seen as pushing gene expression levels from unhealthy states to healthy states, and controllability is a theoretical guarantee that there are possible inputs that can achieve healthy states. Although we found all systems to be controllable, we did find different degrees of controllability. The condition number of the controllability matrix was taken as a measure of degree of controllability and the redox cycle system in mice lung exhibited over 100 times difference in its condition number, suggesting a much higher possibility that unacceptably large inputs are required to move the system into desired states.

Of course much work remains. So far in this report we have only analyzed a small number of dynamical properties while many more remain. Robustness is an important property that some consider an organizing principle of complex biological system [31,32], yet we have not investigated it. There is also the issue of the robustness of estimation. Due to inherent noise in measurements, there are inevitable uncertainties in any parameter estimation. In general, increasing the sample size will increase the reliability of the results for dynamic properties. Another way to deal with this is to obtain confidence intervals for estimated parameter values. However, confidence intervals on individual parameters do not directly translate into confidence intervals for dynamical properties, especially because we have imposed constraints on the parameter space. This should be a topic for further study.

On the issue of scalability, it is known that the number of floating point operations roughly grows to the cubic power of the number of states [23], assuming that the number of states is larger than either the number of inputs or that of outputs. We have implemented our method in Matlab and for the systems studied in this report computation time is around ten minutes on a 1.6 GHz Core Duo laptop, so we expect our program to have no difficulty with a network with dozens of genes. For large systems, we should investigate hierarchical system identification method [33].

## Conclusion

Dynamical properties are considered to be pivotal in determining cellular functions such as apoptosis, cell division, proliferation, etc. [34], and it follows that differential dynamical properties can serve as important indicators for discovering the role of specific biological processes in causing the malfunction of cells. Only by comparing fundamentally different dynamical behaviours between normal and abnormal cells can we begin to untangle the complex interactions and roles of genes in pathogenesis. This will not only add to our understanding of diseases but could also be a step toward effective treatments.

## Methods

### Data sources

To test our method on real-world data, we obtained three data sets: *E. coli *under radiation, mice lung cells exposed to the normal air and a toxin, and mammalian cell lines under the influence of various types of Vpr. They were chosen because they all have time course data of organisms reacting to different perturbations and therefore could embody differential dynamical properties.

Ronen et al. [35] irradiated *E. coli *and used green fluorescent protein (GFP) to obtain the rate of transcription of various genes in the SOS network. Ronen et al. tracked 8 genes of the SOS network as they reacted to different irradiation levels, 5 Jm^-2^and 20 Jm^-2^. Each level had two samples and each sample had 50 time points. They monitored eight genes: uvrD, lexA, umuD, recA, uvrA, uvrY, ruvA, and polB. They performed extensive data pre-processing on the raw data using hybrid Gaussian median filter and polynomial fit for smoothing. They also assumed that the rate of accumulation of GFP was proportional to transcript production, so we shall make the same assumption.

Sciuto et al. [26] measured the effects of carbonyl chloride (phosgene) on mice lung. They exposed the mice to either normal air or phosgene for 20 minutes at a concentration of 32 – 42 mg/m^3 ^and sacrificed some of the mice at each time point. Each time point had 3 samples for air or phosgene and two replications. All experimental data were collected using Affymetrix Mouse 430A oligonucleotide arrays. The raw data were transformed by adding a constant first, and then they performed a log transformation.

Yoshizuka et al. [27] observed the effect of viral protein R (Vpr) on cell cycles. They transfected plasmids that expressed wild type Vpr and mutated Vpr (R73A and R80A) into mammalian cells. The microarrays (Hs Operon V2) containing 22,434 oligonucleotide (60- to 70-mer) spots on a glass slide were used to generate the data. There were three replications for each time point.

Our analysis in this paper was done exclusively on the three data sets above.

### Transfer functions and dynamical properties

A transfer function is a Laplace transform of a linear ordinary differential equation of constant coefficients with zero initial conditions. A single transfer function represents a single-input-single-output (SISO) system and one can obtain a series of transfer functions from a state-space representation of a dynamical system and vice versa [36]. The zeroes are roots of the numerator. The characteristic equation of the transfer function is the denominator equal to zero, and it determines a lot of the dynamical properties of the system. In particular, the roots of the characteristic equation are the poles of the system, which determine the stability of the system and have great influence over other dynamical properties.

### Stability analysis

For discrete linear time-invariant systems, the system is stable (its steady states do not diverge) if and only if all of the eigenvalues of the state transition matrix or all of the poles of all the transfer functions have magnitude less than 1 [7]. For continuous systems the requirement is that all eigenvalues or poles have negative real part. The simplicity of determining stability belies its importance, for it is one of the most important, best analyzed, and best known dynamical property. Feedback control's first task is to ensure stability and robust control spends a great deal of efforts to ensure stability for uncertain models [37,38].

### Root-locus plots

The root-locus method graphically illustrates how the poles of the closed-loop system change as the gain of a pure gain controller is varied. Later it is generalized to show how the roots change as any parameter of the characteristic equation varies. The parameters must be in the form of 1 + *KG*(*s*) = 0 where *K *is the gain (or the parameter), *G*(*s*) is a transfer function, and *s* is a complex variable. The gain is required to be non-negative but this is not a problem because we could just make-*G*(*s*) the new nominal system. We only need two criteria to determine the trajectory

|*KG*(*s*)| = 1

∠*KG*(*s*) = 180° + *k*360°

where *k *is some integer. The root-locus plot lies in the complex plane. The path of roots starts at the open-loop poles and ends at the open-loop zeros, and if part of the path lies on the real axis, then it lies to the left of an odd number of poles [36].

### Controllability

Controllability is a concept central in systems theory. It is about the ability of a system to move from any initial state to any final state with final control in finite time. The controllability matrix is defined as *H *= [*B AB A*^2^*B*⋯] for a linear time invariant system (LTI) of $\dot{x}$ = *Ax *+ *Bu *where *u *is an *m *× 1 vector, *x *an *n *× 1 vector, *A *an *n *× *n *matrix, and *B *an *n *× *m *matrix. If the controllability matrix has full rank, then LTI is controllable; otherwise it is uncontrollable. Another way of saying that a matrix is not full rank is that it is singular, and due to numerical inaccuracy of digital computers and model uncertainty, condition number is used to measure how close to singularity a matrix is. The condition number of a matrix is defined to be ||*H*||·||*H*^-1^|| where ||·|| is any matrix norm. We used 2-norm in this report. The condition number of the controllability matrix can be seen as a measure of the degree of controllability. The larger the condition number is, the greater the inputs are needed to reach a target state, even though reaching nearby states requires no great efforts.

### Unit-step signal and step-response plots

A unit-step signal is a constant signal of strength one. The step response is the output of a dynamical system in response to a unit-step input. The step-response plot graphically gives much information about the dynamical properties of a system. The most important property the step response manifests is stability. A stable system's plot will converge to a steady state while an unstable system will diverge or oscillate. Step-response plots also show settling time, rise time, and percent overshoot. Settling time measures how fast the system achieves the steady state and rise time how quickly the system responds to perturbation. Rise time is defined to be the time for the output to go from 10% to 90% of the steady state. Settling time is defined to be the time for the output to reach and stay within a 2% neighborhood of the steady-state value. Percent overshoot or undershoot is the percentage of the maximum or minimum minus the steady state and divided by the steady state. Rise time is generally associated with the speed of the dynamics, that is, how fast the system responds to inputs, while overshoot and settling time measure how close the transient responses stay within the vicinity of the steady states. They are also inversely related in nature, that is, both rise time and settling time cannot be kept small: decrease in one necessitates increase in the other if nothing else changes. The root-locus technique is one way to use feedbacks to design a closed-loop system with better rise time, better settling time, and better overshoot.

### Parameter estimation

We used expectation-maximization (EM) to estimate the parameters of genetic networks. EM is a well known and well studied method [23,39], and its application to estimating parameters of linear dynamical system has received attention recently from Gibson et al. [23], whose notations we shall follow. We first rewrite

$$\begin{array}{c} x_{t+1}=Ax_{t}+Bu_{t}+w\\ y_{t}=Cx_{t}+Du_{t}+v \end{array}$$

as

$$\left[\begin{array}{c} x_{t+1}\\ y_{t} \end{array}\right]=\left[\begin{array}{cc} A & B\\ C & D \end{array}\right]\left[\begin{array}{c} x_{t}\\ u_{t} \end{array}\right]+\left[\begin{array}{c} w\\ v \end{array}\right]\text{, }$$

and make the following definition for the sake of convenience:

$$z_{t}=\left[\begin{array}{c} x_{t}\\ u_{t} \end{array}\right]\;\;\xi_{t}=\left[\begin{array}{c} x_{t+1}\\ y_{t} \end{array}\right]\text{,}\;\;\;\Gamma =\left[\begin{array}{cc} A & B\\ C & D \end{array}\right]\;\;\Pi =\left[\begin{array}{cc} Q & 0\\ 0 & R \end{array}\right]\text{.}$$

Equation (12) then becomes

$$\xi_{t}=\Gamma z_{t}+\left[\begin{array}{c} w\\ v \end{array}\right]\text{, }\left[\begin{array}{c} w\\ v \end{array}\right]∼N\left(\left[\begin{array}{c} 0\\ 0 \end{array}\right],\Pi \right)\text{.}$$

We shall also denote all the observations (or outputs) as **Y**, all the inputs as **U**, and all the states as **X**. We will add appropriate subscripts if we mean they are up to a certain time step for a particular time course expressed as a superscript. The E-step needs to estimate the conditional expectation

Q(*θ*, *θ'*) = E_*θ'*_[log P_*θ*_(**X**, **Y**|**U**)|**Y**, **U**]

where *θ *is a vector of model parameters and *θ' *is the current estimate of the parameters. First, the likelihood function of the *n*th time series, whose length is *τ*_*n*_, is

$$P_{\theta }(Y_{\tau_{n}},X_{\tau_{n}+1}|U_{\tau_{n}})=P_{\theta }(x_{1})\prod_{t=1}^{\tau_{n}}P_{\theta }(x_{t+1},y_{t}|x_{t},u_{t}),$$

where ${\text{P}}_{\theta }(x_{1})\sim N(\mu ,{\text{P}}_{1})$ and ${\text{P}}_{\theta }\left(\left[\begin{array}{c} x_{t+1}\\ y_{t} \end{array}\right]|x_{t},u_{t}\right)\sim N(\Gamma z_{t},\Pi )$. Expanding equation (16) and taking its logarithm gives

$$\begin{array}{c} -2\log{\text{P}}_{\theta }(Y_{\tau_{n}},X_{\tau_{n}+1}|U_{\tau_{n}})=\log|{\text{P}}_{1}|+{\left(x_{1}-\mu \right)}^{T}{\text{P}}_{1}^{-1}(x_{1}-\mu )\\ +\tau_{n}\log|\Pi |+\sum_{t=1}^{\tau_{n}}{\left(\xi_{t}-\Gamma z_{t}\right)}^{T}\Pi^{-1}(\xi_{t}-\Gamma z_{t}). \end{array}$$

Then we define the following notations for *N *time series:

$$\begin{array}{l} ϒ=\sum_{n=1}^{N}\tau_{n}\text{, }\Phi =\frac{1}{ϒ}\sum_{n=1}^{N}\sum_{t=1}^{\tau_{n}}{\text{E}}_{\theta '}\{\xi_{t}^{n}{\left(\xi_{t}^{n}\right)}^{T}|Y_{\tau_{n}}^{n},U_{\tau_{n}}^{n}\},\\ \Psi =\frac{1}{ϒ}\sum_{n=1}^{N}\sum_{t=1}^{\tau_{n}}{\text{E}}_{\theta '}\{\xi_{t}^{n}{\left(z_{t}^{n}\right)}^{T}|Y_{\tau_{n}}^{n},U_{\tau_{n}}^{n}\}\text{,}\\ \Sigma =\frac{1}{ϒ}\sum_{n=1}^{N}\sum_{t=1}^{\tau_{n}}{\text{E}}_{\theta '}\{z_{t}^{n}{\left(z_{t}^{n}\right)}^{T}|Y_{\tau_{n}}^{n},U_{\tau_{n}}^{n}\}, \end{array}$$

where *τ*_*n *_is the number of time points in the *n*th time series. Taking expectation of equation (17) gives

$$\begin{array}{c} -2Q(\theta ,\theta ')=\log|{\text{P}}_{1}|+\text{trace}\{{\text{P}}_{1}^{-1}{\text{E}}_{\theta '}\{{\left(x_{1}-\mu \right)}^{T}(x_{1}-\mu )\}+ϒ\log|\Pi |\\ +ϒ\text{trace}\left\{\Pi^{-1}\left[\Phi -\Psi \Gamma^{T}-\Gamma \Psi^{T}+\Gamma \Sigma \Gamma^{T}\right]\right\}. \end{array}$$

We need the following quantities for equation (19):

$$\begin{array}{l} {\text{E}}_{\theta '}\{y_{t}x_{t}^{T}|Y_{\tau_{n}},U_{\tau_{n}}\}=y_{t}{\hat{x}}_{t|\tau_{n}}^{T}\\ {\text{E}}_{\theta '}\{x_{t}x_{t}^{T}|Y_{\tau_{n}},U_{\tau_{n}}\}={\hat{x}}_{t|\tau_{n}}{\hat{x}}_{t|\tau_{n}}^{T}+P_{t|\tau_{n}}\\ {\text{E}}_{\theta '}\{x_{t}x_{t-1}^{T}|Y_{\tau_{n}},U_{\tau_{n}}\}={\hat{x}}_{t|\tau_{n}}{\hat{x}}_{t-1|\tau_{n}}^{T}+M_{t|\tau_{n}}, \end{array}$$

where ${\hat{x}}_{t|\tau_{n}}=\text{E}\left[x_{t}|Y_{\tau_{n}},U_{\tau_{n}}\right]$, $P_{t|\tau_{n}}=\mathrm{var}\left[x_{t}|Y_{\tau_{n}},U_{\tau_{n}}\right]$, and $M_{t|\tau_{n}}=\mathrm{cov}\left[x_{t},x_{t-1}|Y_{\tau_{n}},U_{\tau_{n}}\right]$; and they are obtained from the Kalman smoother

$$\begin{array}{l} J_{t}=P_{t|t}A^{T}P_{t+1|t}^{-1}\\ {\hat{x}}_{t|\tau_{n}}={\hat{x}}_{t|t}+J_{t}[{\hat{x}}_{t+1|\tau_{n}}-A{\hat{x}}_{t|t}-Bu_{t}-R^{-1}y_{t}]\\ P_{t|\tau_{n}}=P_{t|t}+J_{t}[P_{t+1|N}-P_{t+1|t}]J_{t}^{\tau_{n}}\\ M_{t|\tau_{n}}=P_{t|t}J_{t-1}^{T}+J_{t}[M_{t+1|\tau_{n}}-AP_{t|t}]J_{t-1}^{T} \end{array}$$

where ${\hat{x}}_{t|t}$, *P*_*t*/*t*_, *P*_*t*|*t*-1 _are calculated from the Kalman filter

$$\begin{array}{l} P_{t|t-1}=AP_{t-1|t-1}A^{T}+Q\\ G_{t}=P_{t|t-1}C^{T}{\left(CP_{t|t-1}C^{T}+R\right)}^{-1}\\ P_{t|t}=P_{t|t-1}-G_{t}CP_{t|t-1}\\ {\hat{x}}_{t|t-1}=A{\hat{x}}_{t-1|t-1}+BU_{t-1}\\ \begin{array}{cc} {\hat{x}}_{t|t}={\hat{x}}_{t|t-1}+G_{t}(y_{t}-C{\hat{x}}_{t|t-1}-DU_{t}), & t=1,...,\tau_{n} \end{array}\\ M_{\tau_{n}|\tau_{n}}=(I-G_{\tau_{n}}C)AP_{\tau_{n}-1|\tau_{n}-1}. \end{array}$$

That constituted the E-step. The M-step, due to the constraints imposed by the network structure, is

[Γ_new_Σ - Ψ] ∘ *M *= 0

Π_new _= {Φ - ΨΓ_new_^*T *^- Γ_new_Ψ^*T *^+ Γ_new_ΨΓ_new_^*T*^} ∘ *I*

where Π_new _and Γ_new _are the updated parameters, and *M *is a constraint matrix of the same size as Γ, so that if an entry of Γ is constrained then it is zero and otherwise one. We also assume all noise covariance matrices are diagonal.

### Higher order dynamics

If we stick with one gene for one state, then the system will only have first order dynamics, which are either exponential decay or exponential growth, associated with all the genes, but because oscillation is widely observed in biology, at least second order dynamics should be available to models of genetic networks. We will give a simple derivation of how to add second order dynamics for the individual nodes of the genetic networks using the principle of continuous to discrete conversion. This is similar to d'Alché-Buc's method [28]. The third or higher order dynamics can be similarly added but we do not make use of it in this report.

We shall focus on one node in genetic networks but the results are easily extrapolated to the entire network. Suppose we have a second order linear differential equation describing the dynamics of a node:

$$\ddot{x}+\lambda_{1}\dot{x}+\lambda_{2}x=\sum_{j}w_{j}z_{j}\text{,}$$

where *x *is the node we are interested in, *z*_*j *_is the *j*th nodes' expression levels, *w*_*j *_its corresponding weights, and *λ*_1 _and *λ*_2 _parameters. Let

$$x_{1}=x,\;\;x_{2}=\dot{x}.$$

Then we get

$$\left[\begin{array}{c} {\dot{x}}_{1}\\ {\dot{x}}_{2} \end{array}\right]=\left[\begin{array}{c} x_{2}\\ \sum_{j}w_{j}z_{j}-\lambda_{1}x_{2}-\lambda_{2}x_{1} \end{array}\right]=\left[\begin{array}{cc} 0 & 1\\ -\lambda_{2} & -\lambda_{1} \end{array}\right]\left[\begin{array}{c} x_{1}\\ x_{2} \end{array}\right]+\left[\begin{array}{cc} 0 & \cdots \\ w_{1} & \cdots \end{array}\right]\left[\begin{array}{c} z_{1}\\ \vdots \end{array}\right]\text{.}$$

If the steps are uniform, then we can represent the derivatives of *x *as

$$\frac{dx}{dt}\approx \frac{\Delta x}{\Delta t}\text{, which becomes }\Delta x=x(k+1)-x(k),$$

where *k *is the time step. The equation (26) then becomes

$$\begin{array}{l} \left[\begin{array}{c} x_{1}(k+1)\\ x_{2}(k+1) \end{array}\right]=\left[\begin{array}{c} x_{1}(k)+x_{2}(k)\\ \sum_{j}w_{j}z_{j}(k)-(\lambda_{1}-1)x_{2}(k)-\lambda_{2}x_{1}(k) \end{array}\right]\\ =\left[\begin{array}{cc} 1 & 1\\ -\lambda_{2} & 1-\lambda_{1} \end{array}\right]\left[\begin{array}{c} x_{1}\\ x_{2} \end{array}\right]+\left[\begin{array}{cc} 0 & \cdots \\ w_{1} & \cdots \end{array}\right]\left[\begin{array}{c} z_{1}\\ \vdots \end{array}\right]\text{.} \end{array}$$

The ones and zeros in equation (28) are fixed except in 1 - *λ*_1 _where the whole term is variable. An interesting observation is that all interactions and inputs should be on the second order term *x*_2_.

## Authors' contributions

Except that HX designed the algorithm and programmed the Matlab code, HX and YC both contributed to the manuscript equally.
